# Effects of *Bifidobacterium longum* Subsp. *infantis* CECT 7210 and *Lactobacillus rhamnosus* HN001, Combined or Not With Oligofructose-Enriched Inulin, on Weaned Pigs Orally Challenged With *Salmonella* Typhimurium

**DOI:** 10.3389/fmicb.2020.02012

**Published:** 2020-08-21

**Authors:** Agustina Rodríguez-Sorrento, Lorena Castillejos, Paola López-Colom, Gloria Cifuentes-Orjuela, Maria Rodríguez-Palmero, José Antonio Moreno-Muñoz, Susana María Martín-Orúe

**Affiliations:** ^1^Servicio de Nutrición y Bienestar Animal, Departament de Ciència Animal i dels Aliments, Universitat Autònoma de Barcelona, Bellaterra, Spain; ^2^Laboratorios Ordesa S. L., Barcelona, Spain

**Keywords:** synbiotic, *Salmonella*, piglet, probiotic, prebiotic, inulin, *Bifidobacterium*, *Lactobacillus*

## Abstract

*Salmonella* is a common causative agent of enteric disease and is developing mechanisms of resistance to antimicrobials. Probiotics, such as bifidobacteria and lactobacilli, and prebiotic fibers are a potential alternative to counteract this pathogen as they have demonstrated effectiveness in preventing its adhesion, reducing intestinal damage, and enhancing the host immune system. Furthermore, the benefits are expected to be potentiated when these compounds are administered together. A trial was performed to evaluate the efficacy of two probiotic strains (*Bifidobacterium longum* subsp. *infantis* CECT 7210 (Laboratorios Ordesa S.L.) and *Lactobacillus rhamnosus* HN001, combined or not with a prebiotic containing oligofructose-enriched inulin, against *Salmonella* Typhimurium. Ninety-six piglets (28 days old) were distributed into 32 pens assigned to 5 treatments: one non-challenged (control diet, CTR+) and four challenged: control diet (CTR−) or supplemented with probiotics (>3 × 10^10^ cfu/kg each strain, PRO), prebiotic (5%, PRE), or their combination (SYN). After 1 week of adaptation, animals were orally challenged with *Salmonella* Typhimurium. Feed intake, weight, and clinical signs were recorded. On days 4 and 8 post-inoculation (PI), one animal per pen was euthanized, and samples from blood, digestive content, and ileal tissues were collected to determine *Salmonella* counts, fermentation products, ileal histomorphology, and serum TNF-α and Pig-MAP concentrations. The effect of the oral challenge was evidenced by animal performance, fecal consistency, and intestinal architecture. Regarding the experimental treatments, animals belonging to the PRO group experienced a faster clearance of the pathogen, with more pigs being negative to its excretion at the end of the study and recovering the impaired ileal villi/crypt ratio more rapidly. Animals receiving the PRE diet showed a lower intestinal colonization by *Salmonella*, with no countable levels (<3 cfu/g) in any of the analyzed samples, and an augmented immune response suggested by serum Pig-MAP concentrations. Treatments including the prebiotic (PRE and SYN) showed similar changes in the fermentation pattern, with an increase in the molar percentage of valeric acid concentration in the colon. The SYN group, however, did not show any of the outcomes registered for PRO and PRE in *Salmonella* colonization or in immunity markers, suggesting the lack of synbiotic action in this animal model. Further research is needed to better understand the complex mechanisms behind these effects.

## Introduction

Salmonellosis is a disease caused by non-typhoidal serotypes of *Salmonella*; after campylobacteriosis, it is the second most common foodborne in the European Union (91,662 human cases reported in 2017; European Food Safety Authority, 2019). It is characterized by symptoms such as an acute onset of fever mainly accompanied by abdominal pain, nausea, and diarrhea. The course of the illness is self-limited, and affected individuals recover without treatment, but it can develop to a serious and life-threatening condition in non-immunocompetent patients, such as children and elderly people (World Health Organization, 2018).

Different serovars of *Salmonella* isolated from humans have been found to be “highly” or “extremely highly” resistant to antimicrobials (European Food Safety Authority, 2019). As a consequence, alternatives to these substances that could potentially fight against these pathogenic bacteria, such probiotics and prebiotics, are of a great interest. Different mechanisms could be behind the positive effects against pathogens reported by different probiotics and prebiotics, but in general terms, all of them would boost the natural mechanisms of colonization resistance (Lawley and Walker, 2013; Fehervari, 2019).

Probiotics have demonstrated efficacy against a multitude of enteropathogens. Some strains of *Lactobacillus* prevent the intestinal damage caused by enterohemorrhagic *Escherichia coli* (Johnson-Henry et al., 2008), and specifically, the HN001 (DR20) strain offers protection against *Salmonella* Typhimurium by stimulating the immune response of the host (Gill et al., 2001). In addition, the genus *Bifidobacterium* plays an important role in the maintenance of gut homeostasis (Tojo et al., 2014), and some strains have also been tested in gastrointestinal pathogen infections. Specifically, *Bifidobacterium longum* subsp. *infantis* CECT 7210 has been proven safe and effective against rotavirus in a murine model (Muñoz et al., 2011) and against enterotoxigenic *Escherichia coli* and *Salmonella* Typhimurium in a piglet model (Barba-Vidal et al., 2017).

Administration of prebiotics also represents a good strategy to fight enteropathogens. Compounds such as inulin and oligofructose reduce the adhesion of pathogens to intestinal epithelium by increasing the bifidobacteria and lactobacilli indigenous population able to use these fibers as growth substrates (Bosscher et al., 2006). In the last years, different authors have proposed the combined use of probiotics and prebiotics to selectively increase the survival and activity of the specific probiotic strains, thereby improving their efficacy. This concept is known as symbiotic therapy (De Vrese and Schrezenmeir, 2008) and has shown promising results against acute diarrhea in children (Yang et al., 2019).

The aim of this work was to evaluate a multistrain probiotic composed of *Bifidobacterium longum* subsp. *infantis* CECT 7210 and *Lactobacillus rhamnosus* HN001, the prebiotic oligofructose-enriched inulin and their synbiotic combination in a weaned piglet model orally challenged with *Salmonella* Typhimurium.

## Materials and Methods

A trial was carried out at the Experimental Unit of the Universitat Autònoma de Barcelona (UAB) and received prior approval (Permit No. CEAAH4026) from the Animal and Human Experimental Ethical Committee of this institution. The treatment, management, housing, husbandry and slaughtering conditions conformed to the European Union Guidelines (Directive 2010/63/EU, *European Commission, 2010)*. All efforts were made to minimize animal suffering.

### Animals, Housing, and Experimental Design

This trial was carried out as Level 2 High-Risk Biosecurity procedure and involved personnel with appropriate training. In total, 96 male piglets [Landrace × Large White] × Pietrain of 28 (±3) days of age, weighting 6.81 (±0.13) kg, were used. All animals were obtained from high-sanitary-status farms from mothers serologically negative to *Salmonella.*

**FIGURE 1:**
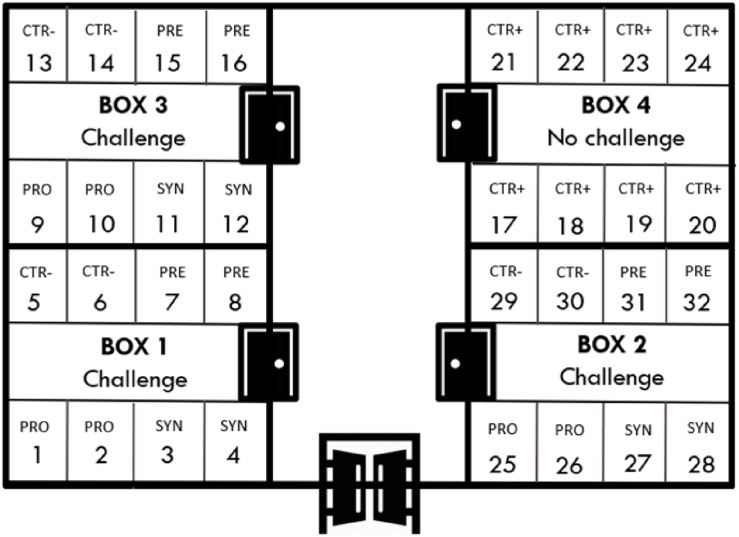
Distribution of experimental treatments in the facilities.

Piglets were transported to the experimental unit located in the UAB, which consisted of four boxes of eight pens each (32 pens, three animals per pen) (see Figure 1). Each 2-m^2^ pen was equipped with a feeder and water nipple to provide feed and water *ad libitum*. All weaning rooms were equipped with an automatic heater and forced ventilation, and each pen had an individual heating light.

At arrival, animals were distributed according to their initial body weight (BW) in order to ensure a homogeneous average BW among treatment groups. The trial was conceived as a completely randomized design that included five experimental groups: non-inoculated control (CTR+) and inoculated control (CTR−), probiotic combination (PRO), prebiotic (PRE) and synbiotic (SYN). The latter four groups were orally challenged and equally distributed in three of the four rooms, while one full room was kept for non-inoculated control pigs. In the challenged rooms, probiotic and synbiotic treatments were distributed within the four pens along one side of the room and the control and prebiotic treatments on the other side of the room, separated by a corridor. Each experimental group had six replicates, except for the non-challenged groups, which had eight replicates.

### Probiotic Strains, Prebiotic Mixture, and Diets

The tested probiotics were *Bifidobacterium longum* subsp. *infantis* CECT 7210, supplied by Ordesa S.L., and *Lactobacillus rhamnosus* HN001 (Danisco USA Inc.) strains. Both were stored in lyophilized form containing 5 × 10^10^ and 3 × 10^10^ colony-forming units [cfu] per gram of product, respectively, in a maltodextrin base. The lyophilized probiotics were daily mixed into the feed for a final dosage of 5.5 × 10^7^ and 3.3 × 10^7^ cfu/g, respectively, totally replacing the feed each day. The viability of the probiotic throughout the day in the dry feed was confirmed prior to the trial. With this aim, one hundred grams of feed belonging to each treatment was analyzed at 0, 6, 12, 24 and 48 h after feed replacement.

The oligofructose-enriched inulin (OF) was in powder form and was manually mixed into the feed up to a final concentration of 5% (w/w); OF was mixed previously to the probiotic in the SYN diet.

Pre-starter diets were formulated in concordance with the nutrient requirement standards for pigs (NRC, 2012) and given in a mashed form. Synthetic amino acids were added to PRE and SYN diets in order to compensate for the possible amino acid dilution produced by prebiotic incorporation: 0.5 g L-valine, 0.9 g L-lysine HCL, 1.2 g DL-methionine, 0.5 g L-threonine, and 0.2 g L-tryptophan per kg of feed. Details regarding the ingredient and chemical composition are given in Table 1.

**TABLE 1:** Ingredients and nutritional composition of the diets.

**Ingredients (g/kg FM)**	**CTR/PRO**	**PRE/SYN**
Maize	206.7	196.3
Wheat	179.4	170.4
Barley 2 row	169.4	160.9
Extruded soybean	148.6	141.2
Sweet whey-powder (cattle)	99.6	94.7
Fish meal	59.8	56.8
Soybean meal 44	79.7	75.7
Whey-powder 50% fat	24.9	23.7
Mono-calcium phosphate	6.8	6.4
Calcium carbonate (CaCO3)	3.9	3.7
L-Lysine HCL	5.3	5.1
Vit-Min Premix*	4	3.8
Sodium chloride (marine salt)	2.5	2.4
DL-Methionine 99	3.9	3.7
L-Threonine	2.8	2.7
L-Tryptophan	0.8	0.8
L-Valine	2	1.9
**Prebiotic**	0	50

**Analyzed composition (g/kg FM)**	**CTR/PRO**	**PRE/SYN**
Dry matter	916.9	917.4
Ashes	48.2	47.7
Crude fat	61.1	58.8
Crude protein	203.4	193.1
Neutral detergent fiber	80.5	82
Acid detergent fiber	25.7	27.1

*Provided per kilogram of complete diet: 10,200 IU vitamin A, 2,100 IU vitamin D3, 39.9 mg vitamin E, 3 mg vitamin K3, 2 mg vitamin B1, 2.3 mg vitamin B2, 3 mg vitamin B6, 0.025 mg vitamin B12, 20 mg calcium pantothenate, 60 mg nicotinic acid, 0.1 mg biotin, 0.5 mg folic acid, 150 mg Fe, 156 mg Cu, 0.5 mg Co, 120 mg Zn, 49.8 mg Mn, 2 mg I, 0.3 mg Se.

### *Salmonella* Strain

The bacterial strain used for the oral challenge was *Salmonella enterica* serovar Typhimurium var. Monophasic (*formula: 4,5,12:i:-, resistance profile: ACSSuT-Ge, Fagotype: U302*), isolated from a salmonellosis outbreak of fattening pigs in Spain, and provided by the Infectious Diseases Laboratory (Ref. 301/99) of the UAB. The preparation of the oral inoculum consisted of 24-h incubation at 37°C in buffered peptone water (Oxoid; Hampshire, United Kingdom) and diluted (1:10) with sterile phosphate buffered saline (PBS) (Sigma-Aldrich; Madrid, Spain). The final concentration of the inoculum was 1 × 10^9^ cfu/mL. Inoculum concentrations were determined prior to inoculation by McFarland standards and were double-plated in Tryptic Soy Agar (TSA) (Liofilche; Italy) on the same day for manual plate counting.

### Experimental Procedure

**FIGURE 2:**
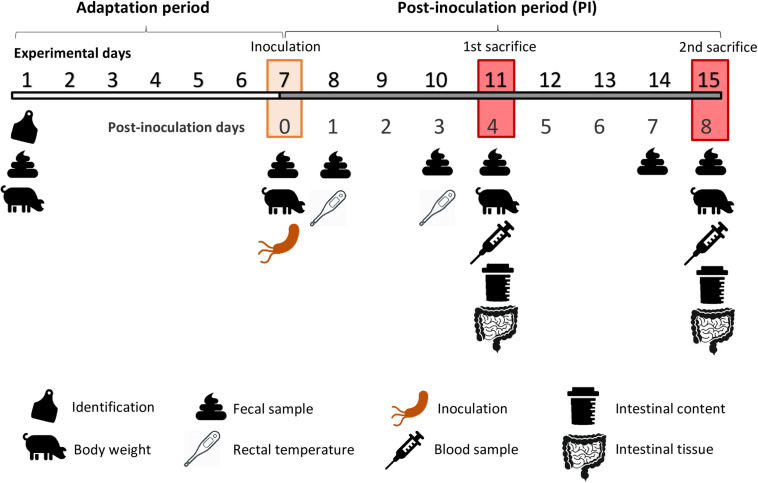
Experimental schedule.

The experimental schedule is depicted in Figure 2. The experimental duration was 15 days. After an adaptation period of 7 days, animals were orally challenged with the pathogen. One animal of each pen was euthanized on days 4 and 8 post-inoculation (PI).

After 1 week of adaptation, the inoculum containing the pathogenic bacterial culture was orally given to each animal of the challenged groups. Each animal received a total volume of 2 mL, corresponding to a 2 × 10^9^ cfu total dose of *Salmonella* Typhimurium. The same amount of sterile broth was administered to non-challenged piglets. To ensure that the animals had a full stomach at the moment of the oral challenge, pigs were starved for a period of 12 hs, and feed was reintroduced 30 min prior to inoculation.

From the challenge onward, animals were checked daily to determine clinical signs and to evaluate their post-inoculation status (i.e., dehydration, anorexia, apathy, general behavior, and fecal score), always by the same person. Fecal score was measured using the following scale: 1 = solid and cloddy, 2 = soft with shape, 3 = very soft or viscous liquid, and 4 = watery or with blood. Rectal temperature was assessed with a digital thermometer (Accuvet, Sanchung City, Taiwan) on days 1 and 3 PI.

To monitor animal performance, individual body weight was registered at arrival and on days 0, 4, and 8 PI. Feed intake was monitored daily. Average daily gain (ADG), average daily feed intake (ADFI), and gain: feed ratio (G:F) were calculated per pen. Mortality rate was also registered, and no antibiotic treatment was given to the animals.

For microbiological analysis, fecal samples were collected aseptically after spontaneous defecation or by digital stimulation. Fecal samples were taken at arrival, the day of the inoculation (0 PI) and on days 1, 3, and 7 PI. Fecal samples were always obtained from the same animal, corresponding to the largest one of each pen at the beginning of the trial (*N* = 32).

On days 4 and 8 PI, one pig per pen was euthanized. On day 4 PI, the selected animal was the one with the medium initial BW, while on day 8 PI, the chosen piglet was the heaviest one of each pen. Animals were euthanized and sequentially sampled during the morning of each sampling day (between 8:00 and 13:00 h). Prior to injection of the euthanasic drug, 10 mL of blood were taken from each animal via venepuncture of the cranial cava vein, using 10-mL blood collection tubes without anticoagulant (Aquisel; Madrid, Spain). Immediately after blood sampling, pigs were administered a lethal dose injection of sodium pentobarbital intravenously (140 mg/kg BW; Euthasol, Le Vet B.V.; Oudewater, Netherlands). Once dead, animals were bled, the abdomen was immediately opened, and the gastrointestinal tract was extracted.

A sample from caecal content was obtained for microbial analysis and kept on ice until analysis within the first 4 h. Subsequently, digesta of ileum and proximal colon were collected and homogenized prior to pH determination with a pH meter (Crison 52–32 electrode, Net Interlab; Barcelona, Spain). Subsamples of ileal and colonic digesta were preserved for different analyses. A set of samples was stored for ammonia determination (NH_3_) (frozen at −20°C in H_2_SO_4_ solution (3 mL of content plus 3 mL of 0.2 N H_2_SO_4_)), and an additional set (∼10 g) for determination of short-chain fatty acids (SCFA) and lactic acid (stored at −20°C).

For the histological study, 1-cm sections from the ileum were removed, opened longitudinally, and washed thoroughly and carefully with 4% formaldehyde solution (Panreac; Castellar del Vallès, Spain) before being fixed by immersion in the same solution.

Blood samples were centrifuged after blood clotting (3,000 × g for 15 min at 4°C), and the serum obtained stored at −20°C.

### Analytical Procedures

Chemical analyses of the diets, including dry matter (DM), ash, crude protein, and diethyl ether extract, were performed according to the Association of Official Agricultural Chemists standard procedures (AOAC International, 1995). Neutral detergent fiber and acid-detergent fiber were determined according to the method of Van Soest et al. (1991).

For microbiological analysis of *Salmonella*, samples were transferred to buffered peptone water (BPW) solution in a concentration of 1:10. Quantitative analysis was performed by seeding serial dilutions of 10^–2^, 10^–4^, and 10^–6^ in xylose-lactose-tergitol-4 plates (XLT-4) (Merck; Madrid, Spain). For the qualitative analysis, samples were incubated in BPW (37°C, 24 h) and 100 μL of the culture were transferred to 10 mL of Rappaport-Vassiliadis for a second incubation (42°C, 48 h) to finally seed them in XLT4 plaques to observe H_2_S-positive colonies.

Short-chain fatty acid and lactic acid analyses were performed using gas chromatography after the samples had undergone acid-base treatment prior to ether extraction and derivatization with N-(tertbutyldimethylsilyl)-N-methyl-trifluoroacetamide plus 1% tert-butyldimethylchlorosilane agent, using the method of Richardson et al. (1989), modified by Jensen et al. (1995).

Ammonia concentration was assed using a gas-sensitive electrode (Hatch Co., Colorado Springs, CO, United States) combined with a digital voltmeter (Crison GLP 22, Crison Instruments, S.A.; Barcelona, Spain). We followed the procedure described by Hermes et al. (2013), which was adapted from Diebold et al. (2004). Samples were diluted (1:2) in 0.16 M NaOH and, after homogenization, centrifuged at 1500 × *g* for 10 minutes. Once the ammonia was released, it was measured in the supernatants as a change in voltage in mV.

Serum concentrations of Tumor Necrosis Factor-α (TNF-α) were determined by Quantikine Porcine TNF-α kits (R&D Systems; Minneapolis, MN, United States), and pig major acute-phase protein (Pig-MAP) concentration was determined by a sandwich-type ELISA (Pig MAP Kit ELISA, Pig CHAMP Pro Europe S.A.; Segovia, Spain) according to the manufacturer’s instructions. Antibodies against *Salmonella* were also assed using an ELISA *Salmonella* Herdcheck (Idexx; Hoofddorp, Netherlands); the established cut-off for positivity in optic density was ≥40%.

For histological study, tissue samples were dehydrated and embedded in paraffin wax: 4-μm thick sections were stained with hematoxylin and eosin. Measurements of 10 different villus-crypt complexes per sample and counting of intraepithelial lymphocytes (IEL), Goblet cells (GC), and number of mitosis of each one were performed with a light microscope (BHS, Olympus; Barcelona Spain), using as guideline the procedure described in Nofrarías et al. (2006).

The concentration of *B. infantis* CECT 7210 was determined by quantitative real-time PCR. To do so, DNA was extracted from the colonic content samples from day 8 PI and the probiotic strain quantified by PCR using the forward (5′-CACAGCGGGCAGATCGGTAT-3′) and reverse primers (5′-CGCCGGTGCCAGTCA-3′) and a TaqMan probe (5′-[6FAM]CCGGTTAGTCCTCTACCGTACGCAAGC[TAM]-3′). The master mix used was “HOT FIREPol Probe qPCR Mix Plus” (Solis BioDyne; Tartu, Estonia).

### Statistical Analysis

The effect of the experimental treatments on the performance and slaughter measurements was determined using free software R v.3.4.4 (R Development Core Team; Franklin Lakes, NJ, United States) and the stats package (R Core Team, 2013) lm function for a one-way ANOVA, with diet as a fixed effect. *Salmonella* spp. count levels and Pig-MAP concentration levels were previously categorized and subsequently analyzed with the fisher.test function for frequency analysis. Pig-MAP concentration levels were categorized considering the normal range (0.3–1 mg/mL), borderline (1–2 mg/mL) and high levels (>2 mg/mL) (Piñeiro et al., 2009). Fisher test was applied to all groups together and also by pairwise. Regarding values for daily fecal consistency data were also assessed using the lme4 package (Douglas et al., 2015) lmer function for an adjusted linear mixed model, with a treatment-by-time interaction term. When treatment effects were established, means were compared by the Tukey–Kramer test (TukeyHSD function). Considering the large number of omnibus test included in this study and with the aim of controlling possible type I errors, *P*-values were also adjusted using the Benjamini–Hochberg procedure.

For all analyzed data, the pen was the experimental unit. The alpha level for the determination of significance for all the analyses was *P* = 0.05. The statistical trends were also considered for *P* values >0.05 and <0.10. Data are presented as means and their residual standard error (RSE) unless otherwise stated.

## Results

The course of the experiment went as expected, without any remarkable incidences. Feed consumption was within the expected values, with piglets receiving the calculated daily amounts of probiotics and prebiotics.

After oral challenge with the pathogenic bacteria, animals presented mild to moderate clinical signs of diarrhea. We registered only one casualty at day 2 PI (CTR− group), and no euthanasia was required.

### Probiotic Viability in Feed and Gastrointestinal Survival

As described in the previous section, the viability of both probiotic strains in animal’s feed was tested. In fact, both of them maintained their viability in the feed for 24 h (*L. rhamnosus* HN001: 7.59 vs 7.57 log cfu/g, *B. infantis* CECT 7210: 7.45 vs 7.33 log cfu/g at 0 and 24 h, respectively), suggesting that animals belonging to PRO and SYN groups were receiving the desired daily amount of probiotics.

Regarding the survival in the gastrointestinal tract of the *B. infantis* CECT 7210 strain, animals belonging to PRO group showed a presence of 5.75 log cfu/g of colonic content, whereas the concentration in those included in SYN group was of 3.96 log cfu/g. No significant difference was found between treatments (*P* = 0.212).

### Performance Parameters

Results for BW, average daily feed intake (ADFI), and average daily gain (ADG) are presented in Table 2.

**TABLE 2:** Effects of experimental treatments on body weight (BW), average daily feed intake (ADFI), and average daily weight gain (ADG).

	**Treatment**					**RSD**	***P*-value**	**Adjusted *P*-value**
	**CTR+**	**PRO**	**PRE**	**SYN**	**CTR−**			
**BW (kg)**								
Initial	6.80	6.76	6.78	6.88	6.81	0.140	0.687	0.777
Final	10.45^a^	9.34^ab^	8.86^ab^	8.21^b^	9.44^ab^	1.067	0.009*	0.045*
**ADFI (g)**								
Adaptation	291.2^a^	242.2^ab^	236.4^ab^	225.2^b^	249.0^ab^	40.16	0.040*	0.131
0–4 PI	395.9^a^	316.1^ab^	287.4^b^	255.2^b^	315.8^ab^	68.55	0.009*	0.045*
4–8 PI	481.5^a^	314.9^b^	299.9^b^	219.1^b^	316.1^b^	103.10	0.001*	0.013*
**ADG (g)**								
Adaptation	206.2	172.3	143.0	150.9	146.5	64.96	0.334	0.477
0–4 PI	274.7^a^	223.7^ab^	153.9^ab^	85.5^b^	222.9^ab^	110.60	0.042*	0.131
4–8 PI	270.0^a^	156.3^ab^	136.4^ab^	38.7^b^	150.3^ab^	97.46	0.003*	0.021*

*Adaptation: pre-inoculation week (days 1–8); 0–4 PI: acute post-inoculation period (days 8-11); 4–8 PI: sub-acute post-inoculation period (days 11–15). CTR+: Non-Inoculated animals receiving placebo; PRO: Inoculated animals receiving the probiotic; PRE: Inoculated animals receiving the prebiotic; SYN: Inoculated animals receiving the synbiotic; CTR−: Inoculated animals receiving placebo. *N* = 6 for all groups except for non-challenged animals, *N* = 8. *P*- values were obtained by an ANOVA, using the linear model procedure in the R software and adjusted by the Benjamini–Hochberg procedure. Means in the same row with different superscripts are different from each other (Tukey’s < *P* 0.05).*

The first effects on average daily feed intake were observed immediately after the oral inoculation. During the acute phase of the infection (days 0–4 PI), PRE and SYN groups also reached significant decreases compared to CTR+ (*P* = 0.049 and *P* = 0.006, respectively), and during the sub-acute phase (days 4–8 PI), all challenged groups showed a reduced feed consumption.

Regarding average daily gain, no significant differences related to diets were found after adaptation nor during the first phase of the infection. However, between days 4 and 8 PI, although all challenged groups showed numerical reductions compared to CTR+, only for the SYN group, this reduction was statistically significant (*P* = 0.001), leading to a lower BW at the end of the experiment (*P* = 0.004).

### Clinical Signs

**FIGURE 3:**
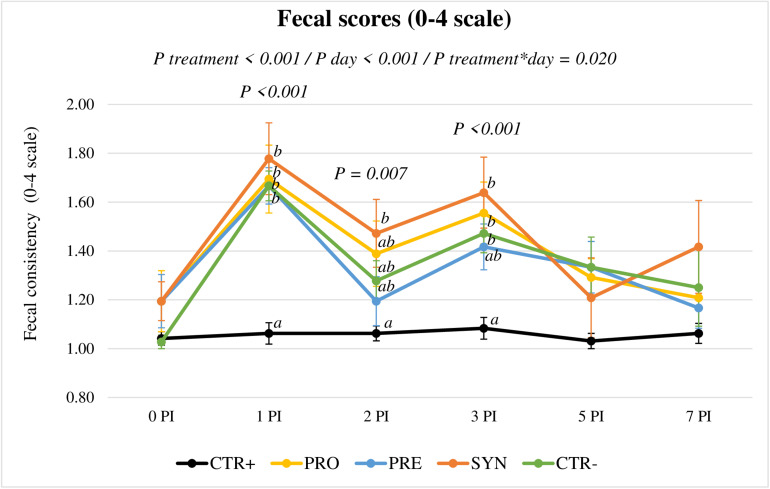
Evolution of average fecal scores for the different experimental groups in the post-inoculation period. CTR+: Non-inoculated animals receiving placebo; PRO: Inoculated animals receiving the probiotic; PRE: Inoculated animals receiving the prebiotic; SYN: Inoculated animals receiving the synbiotic; CTR−: Inoculated animals receiving placebo. *N* = 6 for all groups except for non-challenged animals, *N* = 8. Bars correspond to standard error.

The evolution of fecal consistency along the post-inoculation period is represented in Figure 3. Clinical signs of diarrhea were mild after the challenge, but fecal scores showed a clear increase in all inoculated groups the day after (*P* < 0.001, at day 1 PI). On day 2 PI, the difference to the CTR+ group was only significant for the SYN group (*P* = 0.006), whereas on day 3 PI, all challenged groups except PRE were different from CTR+ (*P* < 0.001). On days 5 (*P* = 0.116) and 7 PI (*P* = 0.140), all inoculated groups recovered, with no significant differences between treatments.

**FIGURE 4:**
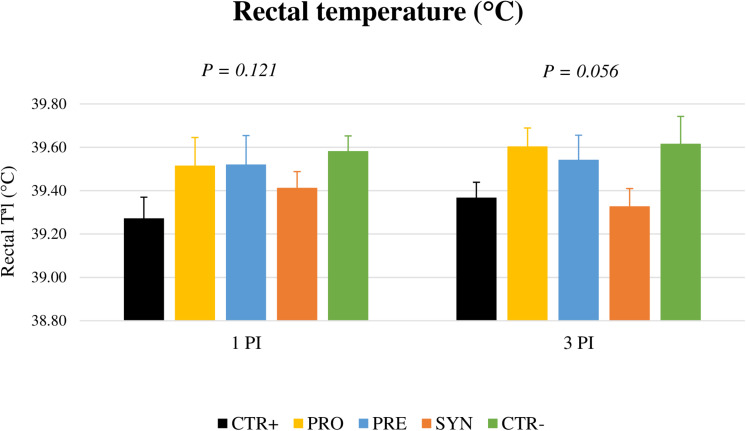
Effects of experimental treatments on rectal temperature on days 1 and 3 post-inoculation. CTR+: Non-inoculated animals receiving placebo; PRO: Inoculated animals receiving the probiotic; PRE: Inoculated animals receiving the prebiotic; SYN: Inoculated animals receiving the synbiotic; CTR−: Inoculated animals receiving placebo. *N* = 6 for all groups except for non-challenged animals, *N* = 8. Bars correspond to standard error.

Febrile response (> 40.5°C) could not be registered in the animals after the challenge (Figure 4). However, on day 3 PI, a trend for an increased rectal temperature was found in the challenged animals except for those receiving the SYN treatment (*P* = 0.056).

### Microbiological Analysis

Serologic analysis confirmed that none of the animals was exposed to the pathogen prior to transfer, with all of them being seronegative at the end of the experimental trial (data not shown).

**FIGURE 5:**
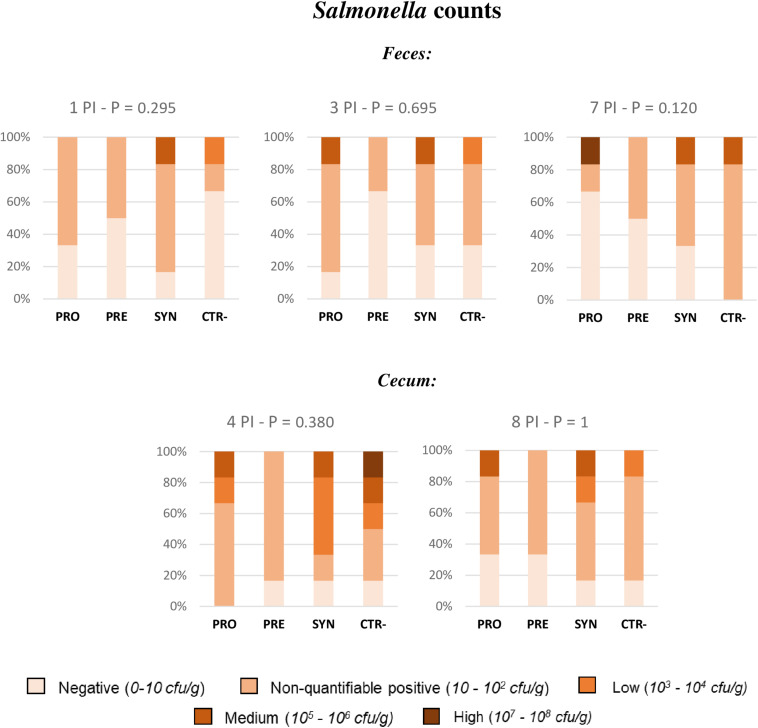
Percentage of animals included in the different fecal and caecal excretion levels of Salmonella. All samples were obtained from the same animal of each pen (with the highest weight) except for the caecal sample on day 4 PI, obtained from the medium-weight animal. PRO: Inoculated animals receiving the probiotic; PRE: Inoculated animals receiving the prebiotic; SYN: Inoculated animals receiving the synbiotic; CTR−: Inoculated animals receiving placebo. *N* = 6 for all groups except for non-challenged animals, *N* = 8. *P*-values were obtained using Fisher’s Exact Test in the R software.

To analyze data from *Salmonella* fecal plate counts, animals were classified into five different levels: high (between 10^7^ and 10^8^ cfu per gram), medium (between 10^6^ and 10^5^ cfu per gram), low (between 10^4^ and 10^3^ cfu per gram), non-quantifiable positive (between 10^2^ and 10 cfu per gram), or negative. This way, the effect of diets was analyzed by Fisher’s exact test, and no significant differences could be found related to experimental treatments (Figure 5).

However, it should be mentioned that when the same test was sectorally applied to compare treatments, it was seen on day 7 PI that a greater percentage of animals receiving the PRO diet became negative to *Salmonella* compared to CTR−animals (65 vs 0%), with significant *P*-values (*P* = 0.028). In addition, when the test was applied to compare treatments in terms of percentage of animals with non-countable *Salmonella* throughout the entire experimental period (negative + very low levels), the only treatment with no animal with countable levels was the PRE treatment, with significant differences when compared to the CTR−group (*P* = 0.013).

### Intestinal Fermentation

The values of intestinal pH, ammonia, lactic acid, and short-chain fatty acid concentrations in ileal and colonic contents for the different experimental treatments are presented in Table 3.

**TABLE 3:** Effects of experimental treatments on ileal and colonic fermentation.

	**PI Day**	**Treatment**					**RSD**	***P*-value**	**Adjusted *P*-value**
		**CTR+**	**PRO**	**PRE**	**SYN**	**CTR−**			
***ILEUM***									
pH	4	6.66	6.50	6.55	6.62	6.61	0.253	0.812	0.842
	8	6.53	6.63	6.86	6.85	6.88	0.299	0.130	0.296
NH_3_ *(mmol/L)*	4	0.59	0.48	0.44	0.54	0.53	0.206	0.715	0.777
	8	0.98	0.99	0.79	1.06	0.70	0.397	0.468	0.616
Lactic acid *(mmol/kg)*	4	9.97	10.48	5.60	3.93	12.26	10.27	0.589	0.718
	8	22.1	22.42	5.38	16.47	5.77	25.10	0.586	0.718
SCFA *(mmol/kg)*	4	6.40	7.12	3.11	3.39	6.27	3.934	0.266	0.474
	8	8.22^a^	6.76^ab^	4.26^ab^	3.09^b^	4.81^ab^	2.954	0.025*	0.104
***SCFA molar ratio (%)***									
Acetic	4	97.3	96.3	94.1	94.7	96.2	4.06	0.623	0.741
	8	96.5	97.3	94.4	85.5	94.9	8.75	0.158	0.330
Propionic	4	3.22	3.36	10.19	6.95	7.20	3.709	0.253	0.474
	8	1.93	1.92	4.55	6.72	4.21	3.544	0.105	0.262
Butyric	4	1.43	2.51	0.83	3.50	2.74	2.085	0.275	0.474
	8	1.52	1.03	1.23	9.26	1.07	7.032	0.286	0.474
***COLON***									
pH	4	5.82^a^	5.65^ab^	5.17^b^	5.42^ab^	5.67^ab^	0.340	0.018*	0.082
	8	5.74	5.76	5.85	5.94	5.92	0.390	0.826	0.843
NH_3_ *(mmol/L)*	4	4.52	8.43	5.31	8.97	9.84	5.517	0.325	0.477
	8	4.66	8.92	10.22	9.07	11.64	6.651	0.369	0.512
Lactic acid *(mmol/kg)*	4	1.16	0.37	1.36	1.41	1.35	1.173	0.695	0.777
	8	0.41	0.38	0.00	0.81	2.15	1.993	0.514	0.660
SCFA *(mmol/kg)*	4	158	152	138	155	147	29.7	0.756	0.804
	8	177	171	165	157	146	41.4	0.706	0.777
***SCFA molar ratio (%)***									
Acetic	4	59.7^a^	57.8^ab^	51.6^b^	53.9^ab^	59.4^a^	3.83	0.002*	0.020*
	8	55.7	58.8	51.6	58.4	61.6	6.03	0.077	0.214
Propionic	4	25.7	24.1	28.3	26.3	24.4	3.51	0.268	0.474
	8	26.9	23.7	29.7	23.8	23.9	4.82	0.145	0.315
Butyric	4	10.8	13.8	14.4	14.6	11.8	3.87	0.297	0.477
	8	12.1	12.9	11.9	11.5	10.3	2.86	0.398	0.538
Valeric	4	2.25^a^	2.59^a^	4.85^b^	4.11^ab^	2.79^ab^	1.246	0.003*	0.021*
	8	2.68^ab^	2.77^ab^	4.81^c^	4.34^bc^	2.23^a^	1.127	0.001*	0.013*
BCFA	4	1.55	1.70	0.86	1.11	1.59	0.593	0.087	0.223
	8	1.56	1.77	1.95	2.04	1.97	0.932	0.863	0.863

*The table includes pH values, ammonia concentration (NH_3_) (mmol/L), lactic acid (mmol/kg of FM), total short-chain fatty acids (SCFA) (mmol/kg of FM), and molar ratio of these SFCA. BCFA = branched-chain fatty acids. CTR+: Non-Inoculated animals receiving placebo; PRO: Inoculated animals receiving the probiotic; PRE: Inoculated animals receiving the prebiotic; SYN: Inoculated animals receiving the synbiotic; CTR−: Inoculated animals receiving placebo. *N* = 6 for all groups except for non-challenged animals, *N* = 8. *P*-values were obtained by an ANOVA, using the linear model procedure in the R software and adjusted by the Benjamini–Hochberg procedure. Means in the same row with different superscripts are different from each other (Tukey’s < *P* 0.05).*

At the ileal level, no significant differences were seen for pH or ammonia concentrations related to the experimental treatments. The concentration of total short-chain fatty acids showed a trend to decrease with the challenge (*P* = 0.025 & adjusted *P* = 0.104). No statistical differences were found in the fermentation profile in terms of molar ratio.

Regarding the colon, no differences related to the experimental treatments were found in ammonia, lactic acid, or total SCFA concentrations. However, on day 4 PI, pH values tended to be lower for the challenged groups, especially for PRE diet compared to CTR+ (*P* = 0.018 & adjusted *P* = 0.082). Although the concentration of total SCFA was not modified by the experimental treatments, there was an impact of the molar ratio for the different fermentation products. On day 4 PI, the PRE group showed a lower percentage of acetic acid than the control groups (*P* = 0.004 and *P* = 0.012 for CTR+ and CTR−, respectively). Moreover, the percentage of valeric acid was numerically higher in those animals receiving de prebiotic (PRE and SYN) at day 4 and 8 PI when compared to the rest of the groups, although statistical significances were only found (*P* < 0.05) when compared to CTR+ (PRE at day 4 and 8 PI) and to CTR− (PRE and SYN at day 8 PI).

### Immune Response

**FIGURE 6:**
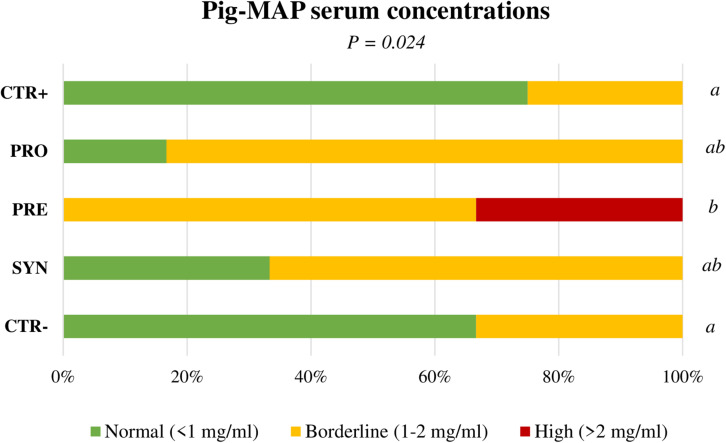
Effect of experimental treatments in serum levels of acute-phase protein Pig-MAP in piglets on day 4 after oral Salmonella challenge. Figure represents frequencies for values between a normal (0.3–1 mg/mL) or abnormal (>2 mg/mL) range. CTR+: Non-Inoculated animals receiving placebo; PRO: Inoculated animals receiving the probiotic; PRE: Inoculated animals receiving the prebiotic; SYN: Inoculated animals receiving the synbiotic; CTR−: Inoculated animals receiving placebo. *N* = 6 for all groups except for non-challenged animals, *N* = 8. *P*-values were obtained using Fisher’s Exact Test in the R software [Piñeiro et al., 2009: normal (≤1 mg/mL), borderline (1–2 mg/mL) high levels (>2 mg/mL)].

The TNF-α did not respond to the challenge and only tended to be higher with the PRE diet at day 4 PI (106.5, 122.6, 147.8, 140.8, and 111.2 pg/mL for CTR+, PRO, PRE, SYN, and CTR−, respectively; *P* = 0.094). Pig-MAP concentrations did not adjust to a normal distribution, and therefore, data were analyzed by frequencies using Fisher’s exact test (Figure 6). In this way, statistical differences were found when PRE treatment was compared to both control groups (CTR+ and CTR−) on day 4 PI, showing a higher percentage of animals with levels above those considered normal (>2 mg/mL) (Piñeiro et al., 2009) (*P* = 0.002 and *P* = 0.006, respectively).

### Intestinal Histological Structure

As depicted in Table 4, 4 days after the challenge, the height of the ileal villi showed a numerical decrease in all inoculated groups, almost reaching the statistical trend value (*P* = 0.033 & adjusted *P* = 0.118). Regarding crypt depth measurements, they were numerically increased with the challenge, albeit only with significant differences for SYN (*P* < 0.001). Consequently, a significantly reduced villous height: crypt depth ratio was found in all challenged groups (*P* < 0.001, adjusted *P* = 0.003) at day 4 PI, with a faster recovery at day 8 PI with the PRO diet, which was the only one not different from CTR+.

**TABLE 4:** Effects of treatments on histomorphological parameters on days 4 and 8 post-inoculation.

	**PI Day**	**Treatment**					**RSD**	***P*-value**	**Adjusted *P*-value**
		**CTR+**	**PRO**	**PRE**	**SYN**	**CTR−**			
Villous height *(μm)*	4	282^a^	229^ab^	213^ab^	184^b^	246^ab^	52.5	0.033*	0.118
	8	259	256	227	227	242	*36.1*	0.320	0.477
Crypt depth *(μm)*	4	228^a^	263^abc^	249^ab^	299^c^	282^cb^	26.2	<0.001*	0.003*
	8	245	266	275	267	282	31.3	0.241	0.474
Villous height: crypt depth ratio	4	1.24^a^	0.88^b^	0.85^b^	0.64^b^	0.87^b^	0.201	<0.001*	0.003*
	8	1.06^a^	0.96^ab^	0.84^b^	0.84^b^	0.85^b^	0.123	0.008*	0.045*
IEL *(Cell no./100 μm)*	4	0.46	0.68	1.02	0.54	0.57	0.776	0.320	0.477
	8	0.50^a^	0.67^ab^	0.98^b^	0.65^ab^	0.45^a^	0.286	0.028*	0.108

*CTR+ – Non-Inoculated animals receiving placebo; PRO- Inoculated animals receiving the probiotic; PRE- Inoculated animals receiving the prebiotic; SYN- Inoculated animals receiving the synbiotic; CTR− - Inoculated animals receiving placebo. *N* = 6 for all groups except for non-challenged animals, *N* = 8. *P*-values were obtained by an ANOVA, using the linear model procedure in the R software and adjusted by the Benjamini–Hochberg procedure. Means in the same row with different superscripts are different from each other (Tukey’s < *P* 0.05).*

Regarding the number of mucosal intraepithelial lymphocytes, on day 8 PI, animals receiving the PRE diet tended to have higher values when compared to CTR+ and CTR−, showing PRO and SYN intermediate levels (*P* = 0.028, adjusted *P* = 0.108). A similar pattern was seen on day 4 PI, although without statistical significance.

## Discussion

One of the main characteristics of probiotics is their ability to antagonize pathogenic bacteria and, thus, to improve host health. As summarized by Markowiak and Śliżewska (2017), the capacity to reach this goal is achieved by four different mechanisms, such as the production of antimicrobial substances (bacteriocins, SCFA, etc.), competition for the adhesion sites in the intestinal epithelium and for nutrients, modulation of the immune system of the host, and blockage of the toxin production by pathogenic bacteria. Among them, *Lactobacillus rhamnosus* HN001 is able to produce bacteriocin-like substances (Aguilar-Uscanga et al., 2013), fact that is not demonstrated for *Bifidobacterium longum* subsp. i*nfantis* CECT 7210 yet, which has just been proven to synthetize a peptide against rotavirus (Muñoz et al., 2011). This experimental trial shows that the probiotic compound formed by *Bifidobacterium longum* subsp. *infantis* CECT7210 and *Lactobacillus rhamnosus* HN001 (DR20) exerted a negative impact on *Salmonella* Typhimurium infection, as it reduced its fecal shedding 7 days after the challenge. Moreover, the animals recovered faster from the intestinal damage produced by the challenge as suggest the improved villi/crypt ratio registered at day 8 PI with this diet. It is not clear, however, which one of the two strains was the main actor of this action or if the effect was, precisely, due to the administration of both strains. Regarding the strain *Lactobacillus rhamnosus* HN001, previous works have demonstrated its effectiveness against *Salmonella* Typhimurium in a murine model by reducing pathogen loads in visceral organs and enhancing the immune system (Gill et al., 2001). This strain has also been considered to be responsible for increasing the blood leucocyte phagocytic activity of mice challenged with *Escherichia coli* O157:H7 (Shu and Gill, 2002). Other strains, such as *Lactobacillus rhamnosus* (GG), can also reduce the levels of *Salmonella* Infantis in the jejunum of inoculated pigs, thereby mitigating intestinal inflammation response caused by this bacterium (Yang et al., 2017). These outcomes are, therefore, consistent with the improved *Salmonella* clearance found in our study in the PRO group.

Regarding *Bifidobacterium longum* subsp. i*nfantis*, benefits against infectious agents have been described. Mice pre-treated with the 35624 strain, in a scenario of a *Salmonella* infection, showed a diminished enterocyte damage and a reduced expression of interleukins IL-8 and IL-10 (Symonds et al., 2012). This down-regulation of pro-inflammatory cytokine is consistent with results reported by O’Mahony et al. (2008), where mice consuming this probiotic showed a decrease in the release of IFN-γ, TNF-α, and IL-10 following CD3/CD28 stimulation by a challenge with *Salmonella* Typhimurium or LPS. In addition, other authors have reported more CD4+CD25+ cells in the spleen, associated with pro-inflammatory cytokine inhibition (Maloy et al., 2003; Scully et al., 2013). More specifically, the tested CECT 7210 strain manifested effectiveness against rotavirus infection in mice (Muñoz et al., 2011), with a good outcome against digestive pathogens (*Salmonella* and ETEC) in piglets (Barba-Vidal et al., 2017). In this case, the probiotic reduced pathogen intestinal colonization and modulated the immune response with an increase in the intraepithelial lymphocytes at ileal level. In the present trial, however, we did not find such an effect on IEL numbers with the PRO treatment, neither in TNF-α nor in Pig-MAP. This suggests that the combined use of these two strains does not result in a clear immunomodulatory activity.

Nonetheless, the inclusion of the prebiotic mixture of inulin and oligofructose in the PRE treatment showed changes in immunity parameters, suggesting a possible immunomodulatory effect for this additive. The PRE diet promoted an increase in the concentration of serum Major Acute-phase Protein (Pig-MAP) and also tended to increment the number of ileal IEL. Pig-MAP is mainly synthetized in the liver when an acute phase response is occurring, representing an unspecific reaction to tissue damage. We assume that the registered increase in Pig-MAP with the PRE diet was a response to a higher damage induced by the *Salmonella* infection; however, with this treatment, we did neither find changes in histomorphological parameters nor in *Salmonella* loads, which were actually lower compared to CTR−. In this regard, it should be remembered that the expression of Pig-MAP is, in a certain way, conditioned by cytokine IL-6, as its presence induces a higher production (González-Ramón et al., 2000). Simultaneously, IL-6 is affected by the ingestion of some prebiotic fibers, such as inulin and oligofructose. In a previous study, β2→1-fructans could induce NF-κB/AP-1 activation (Vogt et al., 2013), which can provoke an up-regulation of the IL-6 gene expression (Libermann and Baltimore, 1990), although the literature data are sometimes contradictory. In this regard, Shukla et al. (2016) reported an increase in interleukin in *Giardia*-infected mice treated with inulin, while Zhang et al. (2018) and Marciano et al. (2015) observed a decrease in the IL-6 concentration in rats administered inulin (3%) and inulin/oligofructose (10%), respectively. This apparent inconsistency between studies could be due to a differential effect of these substances, depending on the dose. In this sense, Song et al. (2018) reported that in chickens, while the use of a low dose of inulin (0.25%) was translated into a reduction of IL-6 gene expression, a high dose (2%) was associated with an increase. However, as we did not determine the levels of this interleukin in our animals, we cannot confirm if the increase observed in Pig-MAP with a 5% inclusion level of inulin + OF was or was not related to a down-regulation of IL-6 expression.

Regarding IEL, these cells are part of the gut-associated lymphoid tissue (GALT), and it is believed that they can have a suppressant activity in the development of oral tolerance (Trejdosiewicz, 1992). There are numerous works supporting the effects of prebiotics (including inulin and OF) on the GALT. Some of these studies have been reviewed by Schley and Field (2002), suggesting different hypotheses about the underlying mechanisms of these effects; however, in all cases, they are mediated by an increase in the lactic acid bacterial population, promoted by prebiotics. The proposed mechanisms include direct contact of lactic acid bacteria (or bacterial products) with immune cells in the intestine, synthesis of short-chain fatty acids by the microbiota, and modulation of mucin production. However, from our results, it is difficult to confirm any of these hypotheses, although in the treatments with the prebiotic, the molar proportion of SCFA was modified to a greater extent. A complementary analysis of the microbiota composition would have helped to clarify the possible role of microbial shifts in the local immune response.

Together with changes in immunity markers, the PRE treatment was also able to limit the colonization of the gut by *Salmonella*, considering that this was the only treatment in which *Salmonella* numbers in the digesta were always below the countable levels. Other authors have also described the potential of non-digestible oligosaccharides to control the presence of this pathogen in the gut by different mechanisms. For example, oligofructose has been shown to reduce *Salmonella enteriditis* counts in the cecum of laying hens (Adhikari et al., 2018) and to diminish *Salmonella* adhesion to HT-29 cells by 50% (Wang et al., 2015), which was directly related to its concentration. Moreover, Kanjan and Hongpattarakere (2017), testing the efficacy of inulin in a proximal colon model, also observed a competitive exclusion of *Salmonella* due to nutrient limitation and antibacterial metabolites produced by stimulated bifidobacteria. In our case, we hypothesize that any of these mechanisms could be behind the lower colonization levels found for the PRE treatment, although we cannot provide evidences.

Despite the beneficial effects of the two-strain-probiotic or the inulin+OF mixture, we could not find any synbiotic behavior when both strategies were combined. No benefits were found in *Salmonella* prevalence in the colon or in the immunity response compared to CTR−. The negative impacts of the challenge on fecal consistency or on the villi/crypt ratio were similar for SYN and CTR−. Despite this, the lower weight gains reported for this treatment during the post-challenge period are remarkable. While in formula-fed infants, a moderate weight gain might be desirable to prevent risk of obesity (Appleton et al., 2018), in challenged animals, it is an unmistakable sign of unwellness. This can be partially explained by the drop in feed intake registered in the post-challenged period, particularly in the acute phase (0–4), which was only significant for the SYN treatment. Nevertheless, the decrease in ADG was higher than expected, suggesting that other factors were involved. A decreased digestibility or profitability of the diet could be associated with an increased transit time of digesta with this treatment. In this regard, the inclusion of inulin-type prebiotics has been reported to be responsible for stool softening and increased defecation frequency (Euler et al., 2005; Kapiki et al., 2007), and in our study, numerical increases were seen in fecal scores few days after challenge with the SYN treatment. However, these differences were not significant when compared to the rest of the challenged groups. An impaired digestibility with the SYN diet could also be due to the decreased ileal villous height registered after the challenge, which was more profound with the SYN treatment in the 0–4 PI period. Nevertheless, similar to fecal consistency, the differences to the remaining challenged groups were not significant. Another explanation for the retarded growth would be a potential overgrowth of the probiotic along the small intestine, boosted by the administration of the prebiotic, resulting in nutrient competition with the host. However, these phenomena would have also taken place during the adaptation week before the challenge, and the results obtained do not support this assumption. Finally, it should not be discarded that the lower gains registered for this treatment would have been a random effect associated with a limited number of replicates for assessing the effects on performance (*N* = 6 pens), even more critical in challenging scenarios when a higher residual variability is expected.

The probiotic and prebiotic combination tested in the present study did not exhibit any synbiotic effect against oral *Salmonella* Typhimurium challenge. Previous studies of other authors evaluating the effect of synbiotic combinations have, contrarily, demonstrated positive results. For example, Baffoni et al. (2011) observed a reduction in campylobacter shedding in chickens challenged with this pathogen and treated with *Bifidobacterium longum* subsp. *longum* and fructo-oligosaccharides. Similarly, Naqid et al. (2015) reduced *Salmonella* Typhimurium excretion in infected pigs by using a combination of *Lactobacillus plantarum* and lactulose. Different outcomes could be due to the distinct synbiotic combinations tested, but also to many other factors, such as diet and prebiotic dose.

The probiotic strains *Bifidobacterium longum* subsp. *infantis* CECT 7210 and *Lactobacillus rhamnosus* HN001 and the prebiotic mixture of inulin, enriched with oligofructose, can provide benefits in a *Salmonella* Typhimurium infection scenario. While the two-strain probiotic appears to speed-up the clearance of the pathogen probably by competitive-exclusion mechanisms, the tested prebiotic mixture reduced colonic colonization possibly by modulation of the local and systemic immune response. However, these desirable effects are not synergistic when the two compounds are administered in a synbiotic combination. Results of this work give insights regarding the safety of the use of these probiotics to fight enteropathogens like *Salmonella* in infants and, moreover, open the possibility of its use in livestock as feed additives to be included in the pre-starter diets. More studies are therefore required to better understand the mechanisms involved and why synergic effects were not seen in the synbiotic combination. A better understanding of the changes induced in the intestinal microbiota, and in its cross-talk with the host, are probably the key for a mechanistic approach.

## Data Availability Statement

The datasets generated for this study are available on request to the corresponding author.

## Ethics Statement

The animal study was reviewed and approved by the Animal and Human Experimental Ethical Committee of Universitat Autònoma de Barcelona (UAB) and its competent authorities (Permit No. CEEAH: 4026 DMAH: 10118). The treatment, management, housing, husbandry and slaughtering conditions conformed to European Union Guidelines (Directive 2010/63/EU, European Commission, 2010). Euthanasia was performed by intravenous injection of sodium pentobarbital. All efforts were made to minimise animal suffering.

## Author Contributions

AR-S participated in the experimental design and was responsible for the animal trial, laboratory analysis, data analysis, and writing. PL-C participated in the experimental design, animal trials, data analysis, and writing. GC-O, JM-M, and MR-P participated in the experimental design and contributed to data analysis and writing. SM-O participated in the experimental design, animal trials, laboratory analysis, data analysis, and writing. All authors contributed to the article and approved the submitted version.

## Conflict of Interest

GC-O, MR-P, and JM-M were employed by Laboratorios Ordesa S. L.

The remaining authors declare that the research was conducted in the absence of any commercial or financial relationships that could be construed as a potential conflict of interest.
